# Quantitative mapping of RNA-mediated nuclear estrogen receptor β interactome in human breast cancer cells

**DOI:** 10.1038/sdata.2018.31

**Published:** 2018-03-06

**Authors:** Giorgio Giurato, Giovanni Nassa, Annamaria Salvati, Elena Alexandrova, Francesca Rizzo, Tuula A. Nyman, Alessandro Weisz, Roberta Tarallo

**Affiliations:** 1 Laboratory of Molecular Medicine and Genomics, Department of Medicine, Surgery and Dentistry “Scuola Medica Salernitana”, University of Salerno, 84081 Baronissi (SA), Italy; 2 Genomix4Life srl, Department of Medicine, Surgery and Dentistry “Scuola Medica Salernitana”, University of Salerno, 84081, Baronissi (SA), Italy; 3 Department of Immunology, Institute of Clinical Medicine, University of Oslo and Rikshospitalet Oslo, 0372 Oslo, Norway

**Keywords:** Breast cancer, Proteomic analysis

## Abstract

The nuclear receptor estrogen receptor 2 (ESR2, ERβ) modulates cancer cell proliferation and tumor growth, exerting an oncosuppressive role in breast cancer (BC). Interaction proteomics by tandem affinity purification coupled to mass spectrometry was previously applied in BC cells to identify proteins acting in concert with ERβ to control key cellular functions, including gene transcription, RNA splicing and post-transcriptional mRNA regulation. These studies revealed an involvement of RNA in ERβ interactome assembly and functions. By applying native protein complex purification followed by nano LC-MS/MS before and after *in vitro* RNA removal, we generated a large dataset of newly identified nuclear ERβ interactors, including a subset associating with the receptor *via* RNA bridging. These datasets will be useful to investigate further the role of ERβ, nuclear RNAs and the other proteins identified here in BC and other cell types.

## Background & Summary

The role of estrogen receptor β (ERβ) in cancer biology is still an open issue, despite its potential use as biomarker and drug target. Extensive *in vitro* and *in vivo* evidences point to an oncosuppressive role of this nuclear receptor in breast and other cancers, where it inhibits carcinogenesis, reduces cancer cell proliferation and tumor growth by exerting a specific control on gene transcription and other regulatory functions of the cell^1,2^. Indeed, ERβ inhibits BC cells proliferation and invasion, both with and without ligand^3–5^, showing an additive effect with antiestrogen^6,7^. Furthermore, loss of ERβ expression in invasive BC, in early stages of ductal tumors^8,9^ and in breast tumorigenesis have been reported^10^, together with a positive prognostic value of the presence of ERβ in cancer cells^11^ that may also be an indicator of their responsiveness to endocrine therapy^12,13^. Although some of the results obtained in clinical samples have been challenged, due to the existence of multiple isoform of this protein and uncertainties concerning the specificity of the available antibodies for its detection in tissue specimens, all these evidences points to a key role of ERβ in BC biology. Finally, the functional role of the receptor in the absence of estrogen, a physiological condition during specific phases of the menstrual cycle, before puberty and in post-menopausal women, when the constitutive activities of ERβ might compensate for the absence of circulating hormones, is of great interest but still poorly understood.

Identification and characterization of the multiprotein complexes involved in the functions of ERβ is a critical step to identify the molecular bases of its signaling in BC cells. Interaction proteomics, combining native protein complexes purification and identification by mass spectrometry, is the gold standard to gain such information, and we and others have been mapping ERβ interactomes of human cells under different experimental conditions^14–19^. By this approach, we recently demonstrated that ERβ can interact with AGO2 in BC cells and that this is mediated by one or more RNAs^19^, suggesting for the first time that RNA plays a role in assembly and/or stabilization of ERβ interactomes, as already shown for other nuclear receptors^20–22^.

**Figure 1:**
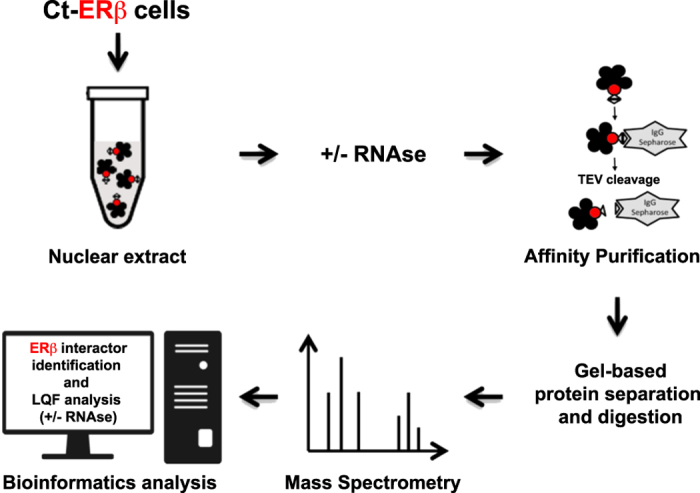
Experimental workflow. Summary of the experimental work-flow applied to generate the protein datasets.

In the present study we generated new ERβ interacting protein datasets by purification of native complexes extracted from C-terminus-tagged expressing ERβ (Ct-ERβ) MCF-7 cell nuclei before and after RNase treatment, followed by label free quantitative proteomics (Fig. 1). Results provide an expanded view of the ERβ nuclear interactome of BC cells, including identification of the protein-protein interactions mediated by RNA, that can now be exploited not only to understand the molecular bases of ERβ activities but also the functions of all other proteins identified.

**Figure 2:**
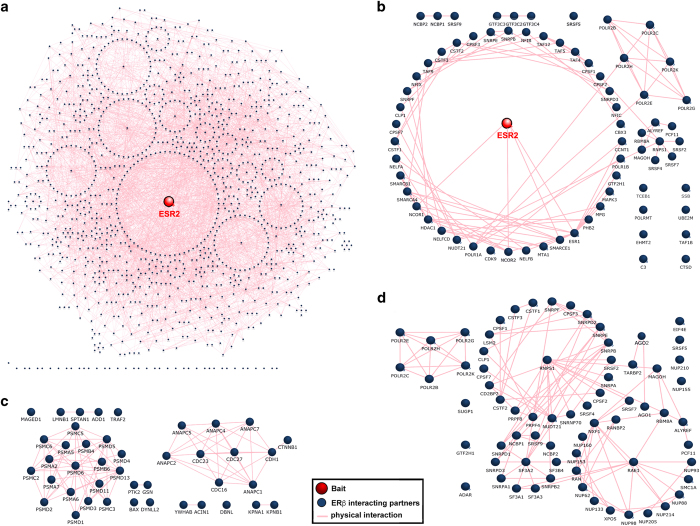
ERβ interaction networks. (**a**) Global network including all the proteins specifically co-purified with ERβ by affinity chromatography and mass spectrometry analysis, showing known associations, reported in protein–protein interaction databases. Sub-networks of ERβ-associated proteins involved in (**b**) transcription and validated nuclear estrogen receptors networks, (**c**) cell death and apoptosis and (**d**) splicing, as obtained by FunRich analysis.

**Figure 3:**
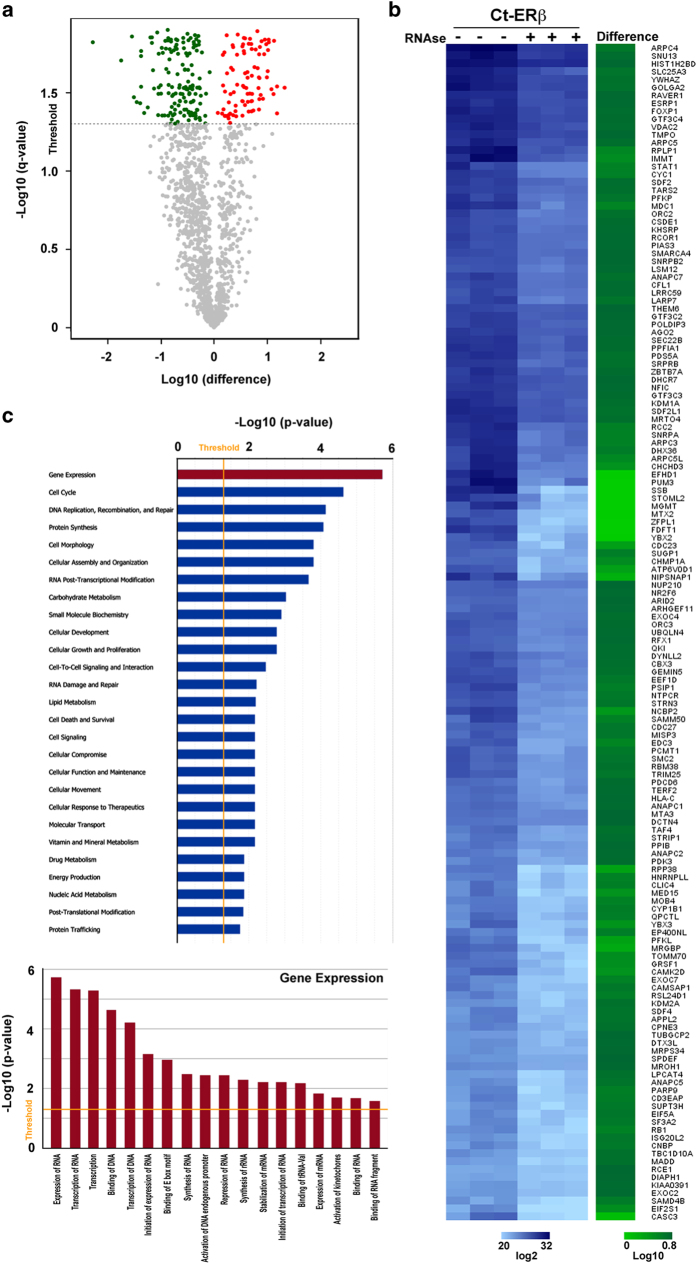
ERβ interactome changes upon RNase treatment. (**a**) Volcano plot summarizing quantitative changes of ERβ-associated proteins upon treatment with RNase. Dotted line (threshold) represents the cut-off (q-value ≤0.05) (**b**) Heatmap of down-represented proteins showing level of LFQ intensities before and after RNase treatment (blu scale). Decrease of protein levels (+ *vs* – RNase) are shown in green scale. (**c**) Functional enrichment analysis by IPA of ERβ-associated proteins whose interaction with the receptor was reduced by RNase treatment (**upper** histogram) and a zoom-in on the Gene Expression functional category (**lower** histogram).

Firstly, ERβ-containing nuclear protein complexes, purified by affinity chromatography (tandem affinity purification (TAP), partial procedure)^23^, were analysed by nano LC-MS/MS, leading to the identification of the largest receptor interactome mapped so far, comprising 1897 specific components, following exclusion of contaminants identified in control samples from ERβ-negative MCF-7 cells processed in the same way, excluding potential contaminants identified in Ct-ERβ samples (e.g. Keratins and Immunoglobulins) (Data Citation 1: ‘Identified proteins’ table). This ERβ interacting network comprises several sub-networks, comprising proteins involved in cellular functions known to be controlled by this receptor, including transcription, cell death and apoptosis and RNA splicing (Fig. 2). RNase treatment was then performed in nuclear extracts from Ct-ERβ cells before nuclear complexes purification and mass spectrometry identification. After discarding the contaminants present in the negative control (Data Citation 1: ‘Identified proteins’ table), and potential contaminants present only in RNAse treated Ct-ERβ samples (e.g. keratins and immunoglobulins, see above), 1453 specific ERβ interactors were identified (Data Citation 1:‘RNA-dependent interactors’ table). A quantitative approach was then applied, by using MaxQuant tool^24^, to identify proteins whose concentration was significantly reduced by pre-treatment with RNase prior to affinity purification, respect to that in untreated samples. Considering the 1472 proteins unequivocally identified and quantified in both Ct-ERβ datasets (untreated and RNase-treated) according to the statistical analyses reported below, the concentration of about 16% of them was significantly (q-value≤0,05) affected by RNA depletion (Fig. 3a and Data Citation 1: ‘MaxQuant analyses’ table). In detail: the concentration of 149 proteins was decreased up to -189 as difference value in RNase-treated *vs* untreated samples, indicating that their association with ERβ is mediated, directly or indirectly, by RNA (Fig. 3b). Concerning the ERβ molecular partners showing increased values after RNase treatment, this could be explained by a more efficient *in vitro* association of these proteins to the receptor moiety following enzymatic treatment, due to a decrease of steric hindrances consequent to removal of RNA and/or associated proteins from the complex.

Interestingly, several of them are known to be involved in the control of gene expression, cell cycle and other functions known to be part of the cellular responses to ERβ (Fig. 3c).

The dataset presented here will be useful to investigate the molecular mechanism of ERβ activity and to design ways to investigate composition and functional roles of macromolecular complexes in BC cell nuclei comprising proteins and RNAs, aiming also at the identification of interactome nodes representing potential drug targets against this, and possibly other, cancers.

## Methods

### Nuclear proteins extraction and treatments

Ct-ERβ^3,14^ and ERβ-negative MCF7 cells (which had been steroid deprived by culturing for 5 days in medium without phenol red and with 5% dextran-coated charcoal treated serum), were harvested by scraping in cold PBS and lysed as previously described^23^. Briefly, cell pellets were resuspended in 3 volumes of hypotonic buffer (20 mM HEPES pH 7.4, 5 mM NaF, 10 μM sodium molybdate, 0.1 mM EDTA, 1 mM PMSF and 1X protease inhibitors cocktail (Sigma Aldrich) and incubated on ice for 15 min. Cytosolic fraction was discarded after adding 0.5% Triton X-100 and spinning for 30 s at 15000 X g at 4 °C. Nuclear pellets were then suspended in 1 volume of nuclear lysis buffer (20 mM HEPES pH 7.4, 25% glycerol, 420 mM NaCl, 1.5 mM MgCl_2_, 0.2 mM EDTA, 1 mM PMSF and 1X protease inhibitors cocktail (Sigma Aldrich), incubated for 30 min at 4 °C rotating and centrifuged for 30 min at 15000 X g at 4 °C. Supernatants were finally recovered, diluted 1:3 with nuclear lysis buffer without NaCl to restore the physiological saline concentration and quantified.

### Purification of ERβ nuclear complexes

IgG-Sepharose beads (GE Healthcare), pre-treated according to the manufacturer’s instructions and equilibrated in TEV buffer (50 mM Tris-HCl pH 8.0, 0.5 mM EDTA, 0.1% Triton X-100, 150 mM NaCl, 1 mM DTT), were added to nuclear protein extracts and incubated for 3 h at 4 °C with gentle rotation, as described earlier^14,16,23,25,26^. Where indicated (see Table 1), 100 μg/ml RNaseA were added to the samples before binding as previously described^19,27^. After incubation, unbound proteins were discarded following centrifugation and the beads were thoroughly washed with 100xVol of IPP150 buffer (20 mM HEPES pH 7.5, 8% glycerol, 150 mM NaCl, 0.5 mM MgCl2, 0.1 mM EDTA, 0.1% Triton X-100) and equilibrated in 30xVol of TEV Buffer in Poly-Prep Chromatography columns (0.8x4 cm, Bio-Rad) at 4 °C. Then 4xBeads Vol of Cleavage Buffer (TEV Buffer containing 1U/μl beads of TEV protease, Invitrogen) were added and two subsequent cleavage reactions were carried out for 2 h and 30 min respectively at 16 °C with gentle shaking. The eluates were then collected, after sedimentation of beads still binding uncut and non-specific proteins.

### Nano LC-MS/MS and Data Analysis

Three biological replicates of partially purified samples from control MCF-7 and from Ct-ERβ cells before and after RNase treatment were analyzed. The protein extracts were precipitated with 10% TCA in Acetone solution, resuspended in Laemmli sample buffer, separated by SDS-PAGE and visualized with silver-staining. The SDS-PAGE lanes were sliced into 6 pieces/each and the proteins were in-gel digested with trypsin (Promega) overnight at 37 °C and eluted as previously described^14,16,23,25,26^. Peptides were desalted and concentrated before mass spectrometry by the STAGE-TIP method using a C18 resin disk (3M Empore). The peptides were eluted twice with 0.1% TFA / 50% ACN, dried and solubilized in 7 μL 0.1% FA for mass spectrometry analysis, that was carried out as previously described^28^. In brief, peptide mixtures were analyzed with an Easy nLC1000 nano-LC system connected to a quadrupole Orbitrap mass spectrometer (QExactive Plus, ThermoElectron, Bremen, Germany) equipped with a nanoelectrospray ion source (EasySpray/Thermo). Liquid chromatography separation of the peptides was performed with an EasySpray column capillary of 50 cm bed length (C18, 2 μm beads, 100 Å, 75 μm inner diameter, Thermo) at 300 nL/min flow rate. Peptides were eluted with a 2-30% gradient of solvent B in 60 min (solvent A: aqueous 0.1% formic acid, solvent B: 100% acetonitrile / 0.1% formic acid). Data-dependent acquisition automatically switched between MS and MS/MS mode and survey full scan MS spectra were acquired from a mass-to-charge ratio (m/z) of 400 to 1,200 with the resolution R=70,000 at m/z 200, after accumulation to a target of 3,000,000 ions in the quadruple. For MS/MS, the ten most abundant multiple-charged ions were selected for fragmentation on the high-energy collision dissociation (HCD) cell at a target value of 100,000 charges or maximum acquisition time of 100 ms. The MS/MS scans were collected at a resolution of 17,500. Target ions already selected for MS/MS were dynamically excluded for 30 s. The resulting MS raw files were submitted for protein identification to Proteome Discoverer 2.1 interface using the Mascot 2.5 search engine. The search criteria for Mascot searches were: trypsin digestion with two missed cleavage allowed, Carbamidomethyl (C) as fixed modification and Acetyl (N-term), Gln->pyro-Glu (N-term Q), Oxidation (M) as variable modifications. The parent mass tolerance was 10 ppm and MS/MS tolerance 0.1 Da. The SwissProt human database was used (August 2016, with 154,660 entries) for the database searches. All of the reported protein identifications were statistically significant (*p*<0.05) in Mascot and filtered in ProteomeDiscoverer for high confident peptides. Only proteins identified in the Ct-ERβ samples and not present in ERβ- control samples prepared in the same way were considered as receptor specific interactors. Additionally, potential contaminants identified only in the Ct-ERβ samples (e.g. Keratins, Immunoglobulins) were discarded and excluded from further analysis (Data Citation 1: ‘Identified proteins’ table). For the experiment performed in presence or absence of RNase the resulting MS raw files were also submitted to the MaxQuant software^24^ version 1.5.7.4 for protein identification and quantitation using the Andromeda search engine (Data Citation 1: ‘RNA-dependent interactors’ and ‘MaxQuant analyses’ tables). Carbamidomethyl (C) was set as a fixed modification and protein N-acetylation and methionine oxidation were set as variable modifications. First search peptide tolerance of 20 ppm and main search error 4.5 ppm were used. Trypsin without proline restriction enzyme option was used, with two allowed miscleavages. The minimal unique+razor peptides number was set to 1, and the allowed FDR was 0.01 (1%) for peptide and protein identification. Label-free quantitation (LFQ) was employed with default settings. The SwissProt human database was used (August 2016, with 154,660 entries) for the database searches. Statistical analysis was performed with the Perseus software (version 1.5.6.0)^29^. Proteins identified in the decoy reverse database, or only by site, were not considered for data analysis. Also, known and potential contaminants provided by MaxQuant and present in the samples were excluded together with all proteins identified by ProteomeDiscoverer/Mascot searches also in the control, ERβ- samples were discarded. The label-free quantification (LFQ) data were log10 transformed, filtered to include only proteins identified and quantified in at least two out of three replicates in at least one experimental group, and missing values were imputed with values representing a normal distribution with default settings in Perseus (generated at 1.8 standard deviations of the total intensity distribution, subtracted from the mean, and a width of 0.3 standard deviations). To find statistically significant differences between the two groups (Sample and RNAse) T-test was performed using permutation-based FDR (0.05 cut-off). The mass spectrometry proteomics data have been deposited to the ProteomeXchange Consortium via the PRIDE^30^ partner repository with the dataset identifier PXD006720 (Data Citation 2) for interaction and quantitative proteomics datasets comprising CTRLs and Ct-ERβ samples before and after RNAse treatment. The list of proteins whose association with the receptor changed after depletion of RNA (Data Citation 1: ‘RNA-dependent interactors’ and ‘MaxQuant analyses’ tables) was compared with that comprising the proteins whose association with the receptor changed upon AGO2 silencing^19^ (Data Citation 3). Results can be found in the figshare repository (Data Citation 1: ‘Comparison of ER beta interactors after either RNA or AGO2 depletion’ table).

### Code availability

The following software and versions were used for quality control and data analysis:

1. For protein identification from raw MS data, Proteome Discoverer software (Thermo) version 2.1 was used: https://tools.thermofisher.com/ with the Mascot 2.5 search engine.
2. For quantitative data analysis, MaxQuant software version 1.5.7.4 with the Andromeda search engine was used: http://www.coxdocs.org/doku.php?id=maxquant:start
3. The SwissProt human database was used (August 2016, with 154,660 entries) as database for protein searches: http://web.expasy.org/
4. For functional interaction networks visualization FunRich v3.0 was used: http://www.funrich.org
5. Functional analysis were performed with IPA software version 01-07: http://www.qiagenbioinformatics.com/products/ingenuity-pathway-analysis
6. Statistical analyses were performed using R www.r-project.org

## Data Records

Data records can be downloaded from our Figshare archive (Data Citation 1). The newly described mass spectrometry proteomics data has been deposited to the ProteomeXchange Consortium via the PRIDE^30^ partner repository with the following dataset identifier: PXD006720 for interaction and quantitative proteomics datasets comprising CTRLs and Ct-ERβ samples before and after RNAse treatment (Data Citation 2).

## Technical Validation

Data shown were obtained from three, independent biological replicates (cell cultures processed independently, each comprising 30×150 mm^2^ dishes at 80% confluency), for both controls and samples, analysed in parallel. The best practice applied in the experimental workflow and technical validations are described below.

**Figure 4:**
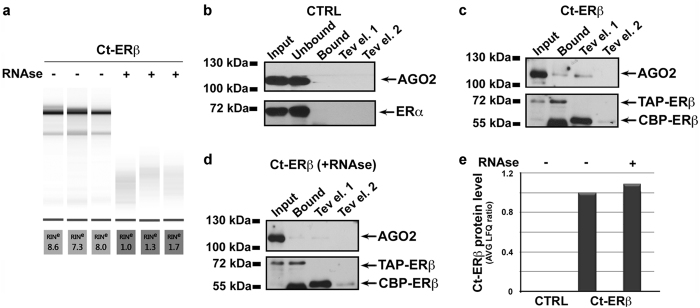
Quality controls of the experimental procedure. (**a**) Electrophoretic quantitation and analysis of RNA extracted from nuclear extracts (starting materials) before and after RNase treatment. Representative western blot analysis of samples from each step of the affinity purification protocol in (**b**) ERβ- (CTRL) and ERβ+ samples before (**c**) and after (**d**) RNase treatment. **CTRL**: Input and Unbound, crude nuclear extracts before and after IgG-Sepharose binding, respectively; Bound, IgG-Sepharose-bound proteins; Tev el. 1 (1st TEV elution) and Tev el. 2 (2nd TEV elution), IgG-Sepharose eluates. **Ct-ERβ**: Input, crude nuclear extracts before IgG-Sepharose binding; Bound, IgG-Sepharose-bound proteins; Tev el. 1 (1st TEV elution) and Tev el. 2 (2nd TEV elution), IgG-Sepharose eluates. (**e**) Quantitation of ERβ concentration in purified samples (TEV elutates) in the absence and presence of RNase. Values represent the LFQ intensity ratios of Ct-ERβ (+ or - RNase) *vs* untreated samples.

Firstly, authenticated cell lines with no more than 5 passages after thawing and mycoplasma-free were used. Affinity purification was performed independently for each replicate processing each control and sample simultaneously in parallel. To quantify the effects of the RNase treatment, RNA was purified from an aliquot of each nuclear extract before TAP and analysed by microfluidic electrophoresis as described^19^ previously (Fig. 4a). Furthermore, aliquots of each sample were kept from each passage and analysed by western blotting to assess presence and integrity of the bait protein ERβ and its known partner AGO2 (ref. 19) in ERβ+ samples and the background in control samples (Fig. 4b-d). Moreover, to exclude that the observed proteome changes were not due to a reduction of ERβ concentration in the starting materials or of its purification efficiency following RNase treatment, ERβ LFQ intensities were compared before and after RNase treatment in Ct-ERβ expressing cells (Figure 4e). The correlation among the triplicate purifications was verified by analysing log2 of LFQ intensities with R version 3.4.2 (see code availability 6), applying the function *co*r from R-package sats v3.4.2 with Pearson correlation method.

Label-free quantitative analysis with MaxQuant was performed to obtain information concerning the impact of RNase on ERβ interactome (differentially purified proteins). Finally, we compared data from the newly obtained datasets with those previously obtained following AGO2 silencing^19^. Data analysis, considering as reference the dataset generated here (PXD006720), confirmed unliganded ERβ interactome composition, with an 85% overlapping between the two datasets. We then compared the proteins whose concentration changed in the purified samples after depletion of either RNA or AGO2, by RNase or siRNA treatment respectively^19^ (Data Citation 1: ‘Comparison of ER beta interactors after either RNA or AGO2 depletion’ table and Data Citation 3). A summary of all data records related to the samples analysed here is reported in Table 1.

## Additional information

**How to cite this article:** Giurato G. *et al.* Quantitative mapping of RNA-mediated nuclear estrogen receptor β interactome in human breast cancer cells. *Sci. Data* 5:180031 doi:10.1038/sdata.2018.31 (2018).

**Publisher’s note:** Springer Nature remains neutral with regard to jurisdictional claims in published maps and institutional affiliations.

## Supplementary Material



## Figures and Tables

**Table 1:** Summary of the protocols and datasets used.

**Sample name (cell line)**	**Protocol 1**	**Protocol 2**	**Protocol 3**	**Treatment**	**Data**
MCF7 (CTRL)_1	Nuclear protein extracts	Tandem Affinity Purification	Nano LC-MS/MS	-	PRIDE PXD006720 (P4883-P4887)
MCF7 (CTRL)_2	Nuclear protein extracts	Tandem Affinity Purification	Nano LC-MS/MS	-	PRIDE PXD006720 (P4889-P4894)
MCF7 (CTRL)_3	Nuclear protein extracts	Tandem Affinity Purification	Nano LC-MS/MS	-	PRIDE PXD006720 (P4896-P4901)
Ct-ERβ (sample)_1	Nuclear protein extracts	Tandem Affinity Purification	Nano LC-MS/MS	-	PRIDE PXD006720 (P4634-4639)
Ct-ERβ (sample)_2	Nuclear protein extracts	Tandem Affinity Purification	Nano LC-MS/MS	-	PRIDE PXD006720 (P4641-P4646)
Ct-ERβ (sample)_3	Nuclear protein extracts	Tandem Affinity Purification	Nano LC-MS/MS	-	PRIDE PXD006720 (P4648-P4653)
Ct-ERβ (sample)_1	Nuclear protein extracts	Tandem Affinity Purification	Nano LC-MS/MS	RNase A	PRIDE PXD006720 (P4655-P4660)
Ct-ERβ (sample)_2	Nuclear protein extracts	Tandem Affinity Purification	Nano LC-MS/MS	RNase A	PRIDE PXD006720 (P4662-P4667)
Ct-ERβ (sample)_3	Nuclear protein extracts	Tandem Affinity Purification	Nano LC-MS/MS	RNase A	PRIDE PXD006720 (P4669-P4674)
Ct-ERβ (sample)_1	Nuclear protein extracts	Tandem Affinity Purification	Nano LC-MS/MS	NT	PRIDE PXD006280 (P5164)
Ct-ERβ (sample)_2	Nuclear protein extracts	Tandem Affinity Purification	Nano LC-MS/MS	NT	PRIDE PXD006280 (P5166)
Ct-ERβ (sample)_3	Nuclear protein extracts	Tandem Affinity Purification	Nano LC-MS/MS	NT	PRIDE PXD006280 (P5168)
Ct-ERβ (sample)_1	Nuclear protein extracts	Tandem Affinity Purification	Nano LC-MS/MS	AGO2 Kd	PRIDE PXD006280 (P5170)
Ct-ERβ (sample)_2	Nuclear protein extracts	Tandem Affinity Purification	Nano LC-MS/MS	AGO2 Kd	PRIDE PXD006280 (P5172)
Ct-ERβ (sample)_3	Nuclear protein extracts	Tandem Affinity Purification	Nano LC-MS/MS	AGO2 Kd	PRIDE PXD006280 (P5174)
